# Household‐Based Epidemiology of Bovine Ixodid Tick Infestation and Associated Risk Factors in Endamehoni, Raya‐Azebo and Enderta Districts, Tigray Region, Northern Ethiopia

**DOI:** 10.1002/vms3.71095

**Published:** 2026-08-05

**Authors:** Girmay Hiluf Asefa, Yohannes Tekle Asfaw, Nigus Abebe Shumuye, Melesew Adisu Abrha, Mebrahtom Gebrelibanos Hiben

**Affiliations:** ^1^ Faculty of Veterinary Science Mekelle University Mekelle Ethiopia; ^2^ Faculty of Pharmacy, College of Health Science Mekelle University Mekelle Ethiopia

**Keywords:** attachment sites, cattle, Ethiopia, hard tick infestation, risk factors, Tigray

## Abstract

**Background:**

Ticks are among the most important ectoparasite of cattle, causing economic losses through reduced productivity, skin damage and the transmission of tick‐borne pathogens. Information on tick epidemiology, community awareness and attachment‐site preferences remains limited in many parts of Ethiopia, including Tigray Region.

**Objectives:**

To determine the prevalence, species composition, risk factors, community knowledge and attachment‐site preferences of bovine ticks in selected districts of Tigray, northern Ethiopia.

**Methods:**

A cross‐sectional study was conducted in three districts of Tigray, northern Ethiopia. A total of 1023 cattle were clinically examined for tick infestation, and collected ticks were identified using standard morphological keys. Household surveys were administered to 422 cattle owners to assess knowledge and control practices. Logistic regression analysis was used to identify factors associated with tick infestation.

**Results:**

Overall, 41.5% (424/1023) of cattle were infested with ticks. A total of 2952 ticks were collected and identified as Rhipicephalus (75.9%), Hyalomma (21.7%) and Amblyomma *variegatum* (2.4%), with Rhipicephalus evertsi evertsi (26.2%), Rhipicephalus decoloratus (25.9%) and Rhipicephalus pulchellus (23.8%) being the predominant species. Tick infestation varied significantly among kebeles. In the multivariable analysis, poor housing (odds ratio [OR] = 10.54), poor body condition (OR = 7.72), herd management practices and cattle breed were significantly associated with infestation, whereas owner literacy was protective (OR = 0.55). Most respondents (91.5%) recognized tick infestation, but only 28.0% associated ticks with disease transmission.

**Conclusions:**

Tick infestation remains widespread among cattle in Tigray and is influenced by animal‐, management‐, and environment‐related factors. Limited awareness of tick‐borne diseases highlights the need for improved farmer education. Integrating community knowledge with species‐specific tick ecology could enhance the effectiveness of targeted tick control strategies.

## Graphical abstract

Tick infestation affected 41.5% of cattle in Tigray, with *Rhipicephalus* species predominating. Infestation was strongly associated with poor housing, poor body condition and management practices, whereas limited awareness of tick‐borne diseases highlights the need for targeted control strategies informed by species‐specific attachment‐site preferences.

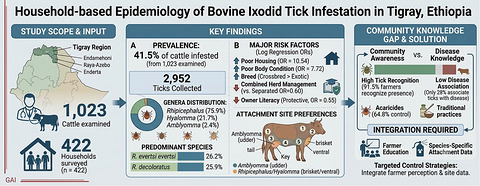

## 1 Introduction

Livestock production is a fundamental component of Ethiopia's agricultural economy and a primary livelihood source for millions of rural households. Ethiopia possesses the largest livestock population in Africa, with the sector playing a crucial role in food security, income generation, asset accumulation, soil fertility improvement and sociocultural practices (Chumburo and Bayou 2021). Despite its immense potential, livestock productivity in Ethiopia remains considerably lower than in many Eastern and sub‐Saharan African countries. This low productivity is mainly attributed to inadequate veterinary services, poor nutrition, limited genetic potential and a high burden of parasitic diseases (Belete and Mekuria 2023).

Among the major parasitic constraints, ticks are regarded as one of the most important ectoparasite due to their extensive distribution and ability to cause significant direct and indirect economic losses. Direct effects include blood loss, skin damage, irritation, reduced weight gain, hide deterioration and toxicosis, all negatively affecting animal health and production efficiency (Wondimu and Bayu 2021; Yadav and Upadhyay 2024). Additionally, ticks act as vectors for bacterial, viral and protozoan pathogens, increasing disease burden, mortality, reduced fertility and veterinary costs (Detamo and Handalo 2020). Chronic infestation may also impair reproductive performance through hormonal disturbances and interference with estrous cycles (Yadav and Upadhyay 2024).

Globally, nearly 80% of the world's cattle population is exposed to tick infestation and tick‐borne diseases, with estimated annual economic losses of $22–$30 billion, particularly in tropical and subtropical regions where smallholder farmers are highly dependent on livestock production. Furthermore, extensive and often indiscriminate use of synthetic acaricides has contributed to acaricide resistance, environmental contamination and food safety concerns, making tick control increasingly difficult and costly (Yenew et al. 2025). Consequently, sustainable integrated tick management strategies are becoming increasingly important worldwide.

In Ethiopia, ticks are widely distributed across different agro‐ecological zones, with prevalence influenced by climate, host‐related factors, husbandry systems and management practices (Gobe et al. 2024). Environmental changes such as deforestation, land degradation, climate variability and increased livestock movement have also contributed to tick proliferation and rising tick‐borne disease risk. Additionally, improper acaricide use and limited veterinary extension services further aggravate the problem (Cardillo et al. 2025). Despite their considerable veterinary and economic importance, ticks have historically received less scientific attention than other arthropod vectors like mosquitoes (Perumalsamy et al. 2024).

The most economically important tick genera infesting livestock belong to the family Ixodidae, particularly *Rhipicephalus*, *Hyalomma* and *Amblyomma variegatum* (Tsolo and Demeke 2024). Ethiopia harbours more than 60 tick species, among which *Rhipicephalus (*Boophilus*) decoloratus* and *A. variegatum* are the most prevalent and economically significant for cattle (Mathewos et al. 2021). Numerous studies across Ethiopia report high cattle tick infestation rates: 68.2% in southern Ethiopia (Belete and Mekuria 2023), 89.9% in Borana pastoral areas (Ayanaa et al. 2021) and 75.8% in Aleltu district (Gobe et al. 2024). Moreover, a recent systematic review and meta‐analysis reported a pooled prevalence of 49.95% in small ruminants across Ethiopia (Girma et al. 2026).

In the Tigray Region, tick infestation is also recognized as a major constraint to livestock productivity and animal health. Previous studies have documented prevalence rates of 90% in Raya‐Azebo district (Hadgu et al. 2018), 71.6% in and around Mekelle (Wegayehu et al. 2023) and 44.7% in goats and 29.5% in sheep in Enderta district (Degefa et al. 2018). Earlier investigations also reported substantial ectoparasite burdens in small ruminants in southern and eastern Tigray (Mulugeta et al. 2010; Barak, 2020). These findings indicate that ticks remain widespread and continue to pose a serious challenge to livestock production systems in the region.

However, most previous studies conducted in Ethiopia, including those in Tigray, were primarily animal‐based cross‐sectional investigations focusing mainly on prevalence estimation and tick species identification. Limited attention has been given to household‐level epidemiological determinants and management‐related risk factors associated with bovine tick infestation. Furthermore, several studies did not incorporate combined analyses of host, environmental and managerial factors influencing infestation dynamics (Fentie et al. 2026; Assefa and Tibebu et al. 2026; Hailu et al. 2024). Comparative epidemiological assessments among different cattle breeds—local, crossbred and exotic—also remain inadequate.

Importantly, comprehensive household‐based epidemiological studies on bovine Ixodid tick infestation are still lacking in Enderta, Enda‐Mehoni and Raya‐Azebo districts of Tigray Region. Although some information is available for Raya‐Azebo district, previous studies were limited in scope, mainly emphasizing prevalence estimation without integrating household management practices, breed susceptibility and multi‐level risk factor analyses. Moreover, no recent integrated epidemiological investigation has evaluated bovine tick infestation across these districts in relation to recent changes in livestock production systems, environmental conditions and accessibility of veterinary services following the socio‐economic disruptions experienced in the region.

Therefore, the present study was designed to generate updated household‐based epidemiological evidence on bovine Ixodid tick infestation and associated risk factors in Enderta, Enda‐Mehoni and Raya‐Azebo districts of Tigray, northern Ethiopia. The findings of this study are expected to provide valuable baseline information for the development and implementation of effective, sustainable and evidence‐based tick control and prevention strategies in the region.

## 2 Materials and Methods

### 2.1 Study Area

The study was conducted in three districts of the southern and south‐eastern zone of Tigray, northern Ethiopia: Raya‐Azebo (12°47′55″ N, 39°38′45″ E), Endamehoni (12°45′ N, 39°30′ E) and Enderta (13°43′0″ N, 39°37′0″ E). These districts were selected due to their high cattle population density and their importance in livestock production systems in the region (Haile et al. 2025; Kelelom et al. 2026; Belay et al. 2021). The districts are also known to experience persistent tick infestations affecting cattle health and productivity, which makes them suitable for epidemiological investigation of tick prevalence and associated risk factors (Hadgu et al. 2018; Degefa et al. 2018).

Ecologically, the study areas represent diverse agroecological zones ranging from lowland to midland and highland environments, which influence tick species distribution, abundance and seasonal dynamics (Hadgu et al. 2019). Such variation in altitude, temperature and humidity is recognized as a key determinant of tick population ecology in tropical livestock systems (Assefa and Tibebu 2026). Furthermore, constraints such as limited access to veterinary services, high costs of acaricides and low farmer awareness of tick control strategies have been reported as major factors contributing to tick infestation persistence in rural Ethiopian cattle production systems (Tesfaye and Abate 2023; Gamal et al. 2023).

#### 2.1.1 Raya‐Azebo Woreda: Geographic and Demographic Profile

Raya‐Azebo comprises three agroecological zones (highland 1.4%, midland 80.6% and lowland 18%) with mean annual rainfall of 610.5 mm (range 351–870 mm) and mean temperatures of 18°C (min.) to 29°C (max.) (Hadgu et al. 2018; Hailu et al. 2024). It has 10 kebeles and 25,991 household heads (12,664 male, 13,328 females). Three kebeles were selected: Tsig'a (2232 households: 1126 males and 1106 females), Wargba (2077 households: 770 males and 1307 females) and Genetie (3326 households: 1565 males and 1761 females) (RARDO 2025).

#### 2.1.2 Enderta Woreda: Geographic and Demographic Profile

Enderta covers ∼93,048 km^2^, bordering Afar, Kilte‐Awulaelo, Hintalo‐Wajirat, Saharti‐Samre and Degua Tembien (Degefa et al. 2018). It has 24 tabias and 41,345 household heads (30,563 male and 10,782 females). Three kebeles were selected: Didba (2444 households: 1637 males and 807 females), Mahbere‐Genet (2547 households: 1662 males and 885 females) and Derg‐Ajen (2170 households: 1671 males and 499 females) (EARDO 2025).

#### 2.1.3 Endamehoni Woreda: Geographic and Demographic Profile

**FIGURE 1:**
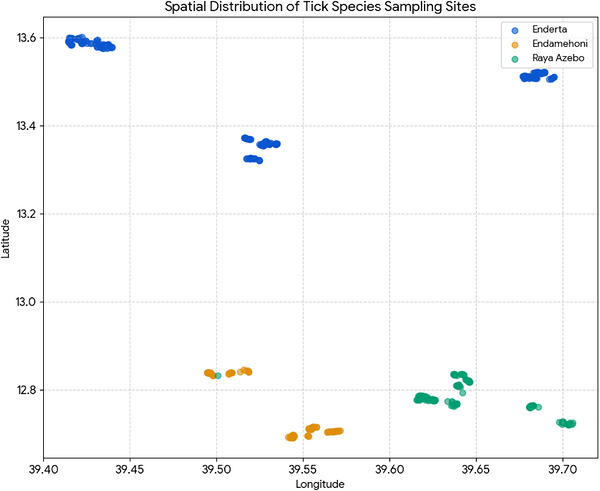
Spatial distribution of tick species sampling sites in Enderta, Endamehoni and Raya Azebo districts, Tigray Region, northern Ethiopia.

Endamehoni borders Ofla (south), Amhara Region (west), Alaje (north) and Raya‐Azebo (east). Temperature ranges 6–32°C, with bimodal rainfall (Kiremt: June–September; Belg: January–March) (Belay et al. 2021). Agroecological zones: highland 65%, temperate 30% and lowland 5%. It has 10 tabias and 16,643 households (8231 male and 8412 females) (Endamehoni Agricultural Office 2025) (Figure 1).

### 2.2 Study Design

A cross‐sectional study was conducted from October 2024 to June 2025 to determine tick infestation prevalence, identify tick genera/species, examine attachment sites and evaluate risk factors (age, breed, season, body condition, sex and location). Each animal was inspected by counting ticks from anatomical regions (scrotum/teats/udder, tail, head, dewlap, brisket, lateral/ventral body surfaces). Collected ticks were preserved in 70% ethanol for identification.

### 2.3 Questionnaire‐Based Survey Using Kobo Toolbox

A semi‐structured questionnaire was developed in English, translated into Tigrigna and digitized using Kobo Toolbox for real‐time data collection and GPS tracking of households. Trained enumerators conducted face‐to‐face interviews after pre‐testing the questionnaire outside the study area.

### 2.4 Sampling Methods and Techniques

A multistage sampling method was used. Three woredas were purposively selected based on livestock density, accessibility and reported tick prevalence. Within each woreda, three kebeles were purposively selected (Enderta: Didba, Derg‐Ajen, Mahbere‐Genet; Raya‐Azebo: Tsiga, Wargba, Genetae; Endamehoni: Tahtay‐Haya, Tsibet, Meswa'ti). Cattle‐owning households were identified with local extension personnel, then 422 households were selected by simple random sampling: Enderta (Didba = 71, Derg‐Ajen = 64 and Mahbere‐Genet = 74); Raya‐Azebo (Tsiga = 35, Wargba = 35 and Genetae = 56); Endamehoni (Tahtay Haya = 33, Tsibet = 32 and Meswa'ti = 22). All cattle in selected households not treated with acaricide in the prior 2 weeks were included (total 1023 cattle).

### 2.5 Sample Size Determination

No prior household‐level tick prevalence data existed for the study area. Using Cochran (1977) formula with 95% confidence, 5% precision and 50% expected prevalence, initial sample size was 384 households. Adding 10% for non‐response gave 422 households, which were enrolled.

### 2.6 Sampling and Sample Processing

Age categories are young (1–3 years), adult (3–10 years) and old (>10 years) (Ethiopian Ministry of Agriculture 2025). Body condition score (BCS) was assessed visually and by palpation (M. Nicholson and T. Butterworth 1986): poor—extreme emaciation, prominent spinous/transverse processes; medium—visible ribs, minimal fat, slightly palpable spinous processes; good—well‐developed fat, ribs not visible, processes not easily palpable.

### 2.7 Tick Sampling Procedure and Identification

Ticks were collected from naturally infested cattle. Freshly detached ticks were placed in ventilated plastic cups and transported to Mekelle University Parasitology Laboratory. Each container was labelled with date, kebeles, animal species, sex, age, BCS, attachment site and management system. Identification used stereoscopic microscopy and standard morphological keys (Waktole et al. 2018): mouthparts, scutum shape/extension, leg colouration, festoons, punctations, marginal spots, posterior grooves and adanal shields.

### 2.8 Data Quality Control and Analysis

Data were collected via Kobo Toolbox with built‐in validation rules, skip logic and range constraints. Exported data (Microsoft Excel) were cleaned (duplicates removed, inconsistencies corrected and missing values handled) and imported into SPSS version 27 for analysis.

## 3 Results

### 3.1 Overall Prevalence of Tick Species

Out of 1023 cattle examined, 425 (41.5%) were infested with ticks. A total of 2952 ticks were collected and identified as three genera: *Rhipicephalus* (75.9%), *Hyalomma* (21.7%) and *Amblyomma* (2.4%). The most predominant species was *Rhipicephalus evertsi evertsi* (26.2%), followed by *R. decoloratus* (25.9%) and *Rhipicephalus pulchellus* (23.8%) (Table 1).

**TABLE 1:** Distribution of tick genera and species identified from infested cattle (*n* = 425).

Tick genus	Tick species	No. of ticks	Proportion of total ticks (%)
Rhipicephalus	R. evertsi	774	26.2
	R. decoloratus	766	25.9
	R. pulchellus	702	23.8
Subtotal		2242	75.9
Hyalomma	H. truncatum	628	21.3
	H. marginatum rufipes	12	0.4
Subtotal		640	21.7
Amblyomma	A. variegatum	70	2.4
Total		2952	100.0

### 3.2 Distribution of Ixodid Tick Genera by Sex and Infestation Patterns

A total of 2952 Ixodid ticks were collected and identified from the study districts. The genus *Rhipicephalus* was the most predominant (75.9%), followed by *Hyalomma* (21.7%) and *Amblyomma* (2.4%). Across all genera, male ticks were more frequently collected than females.

Mixed‐species infestations were more common than single‐species infestations for all genera, indicating frequent co‐infestation of cattle by multiple tick species in the study area (Table 2).

**TABLE 2:** Distribution of Ixodid tick genera by sex and infestation patterns.

Genus	Female	Male	F:M ratio	Single tick species	Mixed tick species	Total (% of total)
Rhipicephalus	685	1557	1:2.27	782	412	2242 (75.9)
Hyalomma	87	553	1:6.36	142	236	640 (21.7)
Amblyomma	12	58	1:4.83	8	37	70 (2.4)
Total	784	2168	1:2.77	937	685	2952 (100)

### 3.3 Species‐Level Distribution of Ixodid Ticks by Sex and Infestation Pattern

At the species level, R. evertsi evertsi was the most prevalent species (26.2%), followed by R. decoloratus (25.9%) and R. pulchellus (23.8%). Among Hyalomma species, H. truncatum accounted for 21.3%, whereas A. variegatum (2.4%) and Hyalomma marginatum rufipes (0.4%) were less frequently observed. Overall, male ticks predominated across most species, with a female‐to‐male ratio of 1:2.77. Single‐species infestations were most frequently observed in R. decoloratus, followed by R. evertsi evertsi and R. pulchellus, whereas mixed‐species infestations were also commonly recorded across all species (Table 3).

**TABLE 3:** Species‐level distribution of Ixodid ticks by sex and infestation patterns.

Tick species	Female	Male	F:M ratio	Single tick species	Total (% of total)
Rhipicephalus evertsi evertsi	168	606	1:3.61	270	774 (26.2)
Rhipicephalus decoloratus	366	400	1:1.09	311	766 (26.0)
Rhipicephalus pulchellus	151	551	1:3.65	201	702 (23.8)
Hyalomma truncatum	75	553	1:7.37	147	628 (21.3)
Amblyomma variegatum	12	58	1:4.83	8	70 (2.4)
Hyalomma marginatum rufipes	12	0	N/A	0	12 (0.4)
Total	784	2168	1:2.77	937	2952 (100)

*Note*: Tick species are presented in scientific names (italicized for academic consistency). **Sex** represents the total number of male and female ticks identified. Infestation pattern shows the number of animals infested with either a single species or multiple tick species (mixed). Percentages are calculated on the basis of the total number of identified ticks (*N* = 2952).

### 3.4 Attachment Site Distribution of Tick Species Infesting Cattle

Marked variation in attachment site preference was observed among tick species. Overall, the lateral/ventral body region and dewlap/brisket were the most frequently infested sites across most species. A. variegatum showed a strong preference for the teat/udder region, while R. decoloratus was more evenly distributed across body sites, particularly the tail/head and teat/udder regions. Other tick species showed varying but consistent site preferences, with relatively low infestation observed on whole body and other less accessible regions (Tables 4 and 5).

**TABLE 4:** Attachment site distribution of tick species infesting cattle (*n* = 2952).

Attachment site	Rhipicephalus *pulchellus*	Rhipicephalus evertsi evertsi	Hyalomma truncatum	Rhipicephalus decoloratus	Amblyomma variegatum	Hyalomma rufipes	Total
Lateral/Ventral	847 (34.4%)	742 (32.6%)	815 (41.9%)	164 (28.2%)	62 (23.8%)	28 (28.6%)	2658
Teat/Udder	319 (13.0%)	279 (12.3%)	150 (7.7%)	103 (17.7%)	104 (39.8%)	15 (15.3%)	970
Tail/Head	448 (18.2%)	508 (22.3%)	299 (15.4%)	143 (24.6%)	40 (15.3%)	20 (20.4%)	1458
Dewlap/Brisket	778 (31.6%)	698 (30.7%)	649 (33.4%)	158 (27.1%)	50 (19.2%)	30 (30.6%)	2363
Whole body	20 (0.8%)	10 (0.4%)	0 (0%)	4 (0.7%)	0 (0%)	0 (0%)	34
Others	50 (2.0%)	40 (1.8%)	30 (1.5%)	10 (1.7%)	5 (1.9%)	5 (5.1%)	140
Total by site	2462	2277	1943	582	261	98	7623^a^

^a^ Some ticks were counted in multiple attachment sites; thus, species totals exceed the overall tick count (*n* = 2952).

**TABLE 5:** Socio‐economic characteristics of respondent cattle‐owning households in three districts of Tigray.

Variable	Category	Frequency (*n*)	Percentage
Zone	South‐eastern	209	49.53
	Southern	213	50.47
	Total	422	100.00
Woreda	Endamehoni	87	20.62
	Enderta	209	49.53
	Raya Azebo	126	29.86
	Total	422	100.00
Tabia (Kebelle)	Derg‐Ajen	64	15.17
	Didba	71	16.82
	Genetie	56	13.27
	Mahbere Genet	74	17.54
	Meswati	22	5.21
	Tahtay Haya	33	7.82
	Tsibet	32	7.58
	Tsig'a	35	8.29
	Wargba	35	8.29
	Total	422	100.00
Religion	Islam	15	3.55
	No religion	1	0.24
	Orthodox	406	96.21
	Total	422	100.00
Education level	No education	224	53.08
	Basic literacy	76	18.01
	Primary and junior	93	22.04
	Secondary and above	29	6.87
	Total	422	100.00
Gender	Female	190	45.02
	Male	232	54.98
	Total	422	100.00
Age category	18–35	84	19.91
	36–59	263	62.32
	Above 60	75	17.77
	Total	422	100.00

### 3.5 Variation in Tick Species Prevalence by Risk Factors in Three Districts of Tigray

Chi‐square analysis revealed significant variation in tick prevalence across several risk factors. Prevalence differed significantly among kebeles (*χ* ^2^ = 53.81, *p* < 0.001), with the highest infestation recorded in Tahtay Haya (66.7%) and Wargba (56.2%) and the lowest in Tsibet (12.5%).

Breed was also significantly associated with tick infestation (*χ* ^2^ = 48.75, *p* < 0.001), with local breeds showing the highest prevalence (50.6%) compared to exotic breeds (23.3%). Body condition showed a strong association with infestation (*χ* ^2^ = 170.62, *p* < 0.001), where cattle with poor body condition had the highest prevalence (72.3%).

Housing condition was highly significant (*χ* ^2^ = 241.32, *p* < 0.001), with higher infestation in poorly housed cattle (67.8%). Herd type (*χ* ^2^ = 27.1, *p* < 0.001) and management system (*χ* ^2^ = 28.08, *p* < 0.001) also showed significant effects, with higher prevalence in mixed herds (54.7%) and extensive production systems (46.1%).

Significant differences were also observed by zone (*χ* ^2^ = 7.12, *p* = 0.008) and woreda (*χ* ^2^ = 10.21, *p* = 0.006), with higher prevalence in lowland areas of Raya‐Azebo. Owner education (*χ* ^2^ = 22.95, *p* < 0.001) and gender (*χ* ^2^ = 8.06, *p* = 0.005) were also significantly associated with tick infestation, whereas age and sex of cattle were not statistically significant (*p* > 0.05) (Table 6, Figure 2).

**TABLE 6:** Tick prevalence variation in the studied districts and association between selected risk factors and cattle tick prevalence in the study area.

Study areas or risk factor	Category	*N* (examined)	*N* (positive)	Prevalence (%)	*χ* ^2^	*p* value
Zone	South‐eastern	532	200	37.6	7.123	0.008
	Southern	491	225	45.8		
Woreda	Endamehoni	157	63	40.1	10.208	0.006
	Enderata	532	200	37.6		
	Raya Azebo					
Kebelle	Derg Ajen	129	42	32.5	53.81	<0.001
	Didba	233	86	36.9		
	Genetie	159	76	47.8		
	Mahbere Genet	170	72	42.4		
	Meswati	38	16	42.1		
	Tahtay Haya	60	40	66.7		
	Tsibet	59	7	12.5		
	Tsig'a	86	36	41.9		
	Wargba	89	50	56.2		
Altitude	Midland	662	305	46.1	18.838	<0.001
	highland	361	120	33.9		
Breed	Cross	276	108	39.1	48.75	<0.001
	Exotic	223	52	23.3		
	Local	524	265	50.6		
Sex	Female	532	207	38.9	3.168	0.075
	Male	491	218	44.4		
Age	Adult	465	198	42.6	3.94	0.139
	Old	167	78	46.7		
	Young	391	149	38.1		
Body condition	Good	203	33	16.3	170.62	<0.001
	Medium	542	191	35.2		
	Poor	278	201	72.3		
Herd type	Mixed	278	152	54.7	27.1	<0.001
	Separated	745	273	36.6		
Management system	Extensive system	749	345	46.1	28.076	<0.001
	Semi‐intensive system	199	63	31.7		
	Intensive System	6	1	16.6		
	All types of management system	58	11	18.9		
Housing condition	Good	183	18	9.8	241.32	<0.001
	Medium	390	102	26.2		
	Poor	450	305	67.8		
Gender (HH)	Female	433	202	46.7	5.778	8.062
	Male	590	223	37.8		
Age (HH)	18–35	172	84	48.84	5.778	0.056
	36–59	663	259	39.06		
	≥60	188	82	43.62		
Education level	No education	527	248	47.05	22.947	<0.001
	Basic literacy	192	55	28.65		
	Primary and junior	237	89	37.55		
	Secondary and above	67	33	49.25		

FIGURE 2Bar chart showing the prevalence of cattle tick infestation across selected animal, management and owner‐related risk factors in the study area: (a) prevalence of cattle tick infestation by altitude; (b) prevalence of cattle ticks by breed; (c) prevalence of cattle ticks by body conditions; (d) prevalence of cattle ticks by management system; (e) prevalence of cattle Ticks by herd type; and (f) prevalence of cattle ticks by housing conditions.

**Figure:**
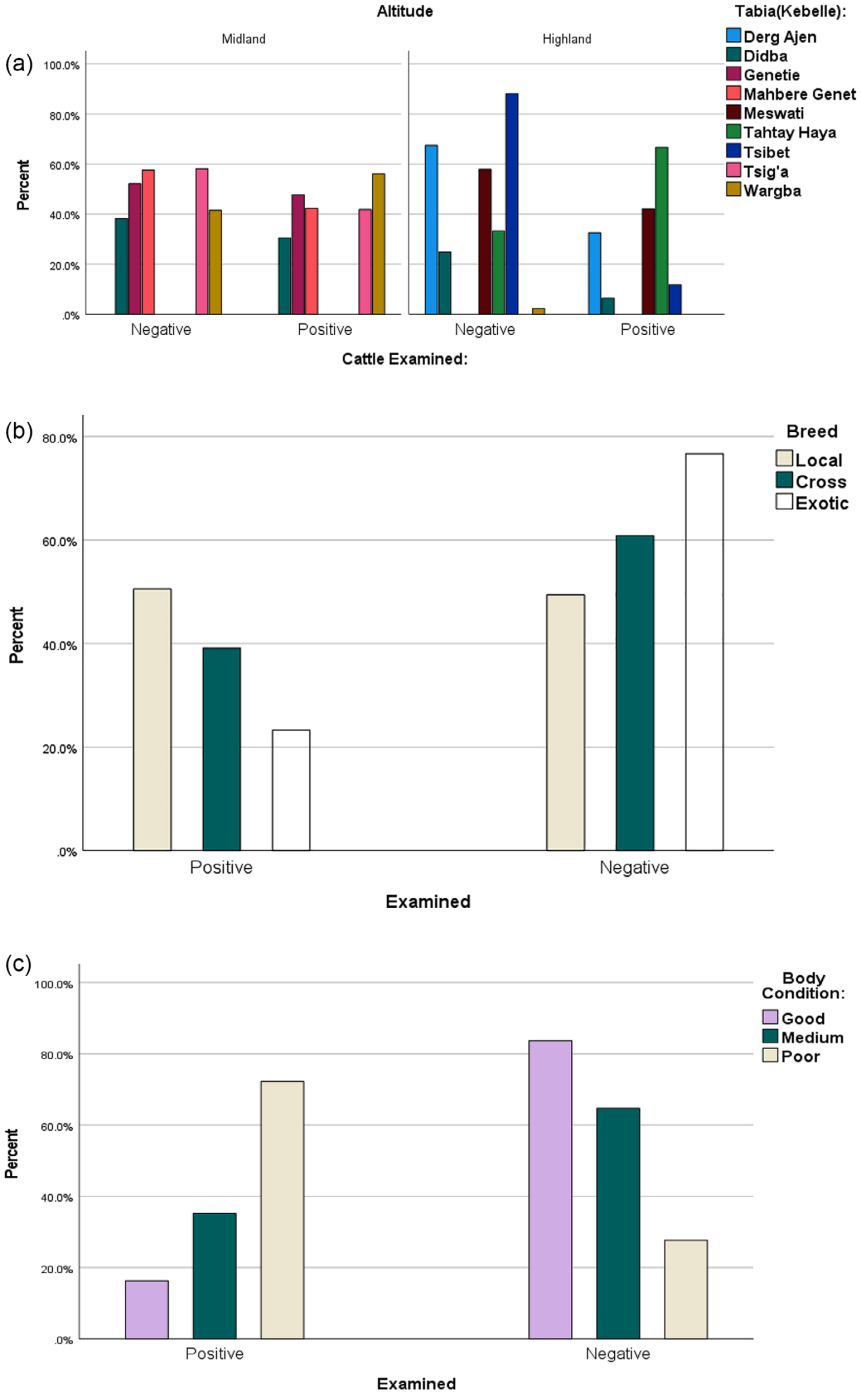

**Figure:**
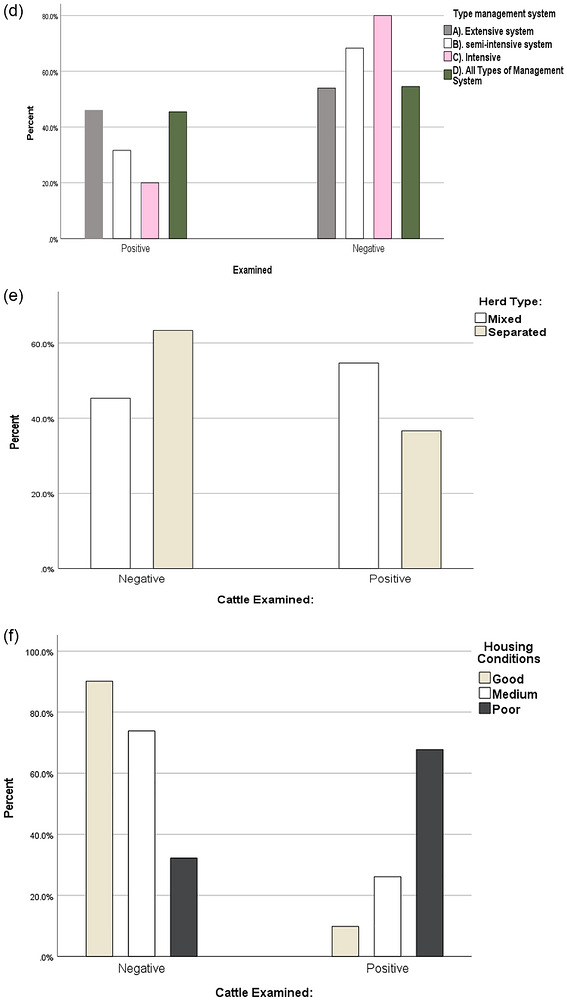

### 3.6 Multivariable Logistic Regression Analysis of Different Risk Factors With Tick Infestation in Cattle (Tables 7A and B)

Multivariable logistic regression analysis identified several significant predictors of tick infestation in cattle, and the model showed acceptable fit and predictive performance. Cattle in Tahtay‐Haya had significantly higher odds of infestation compared to the reference kebeles (Derg‐Ajen) (AOR = 3.74; 95% confidence interval [CI]: 1.62–8.65; *p* = 0.002), whereas cattle in Tsibet had significantly lower odds of infestation (AOR = 0.26; 95% CI: 0.09–0.72; *p* = 0.010).

BCS was strongly associated with infestation. Cattle with poor body condition had markedly higher odds of infestation (AOR = 7.72; 95% CI: 4.58–13.00; *p* < 0.001), followed by those with medium body condition (AOR = 2.12; 95% CI: 1.32–3.41; *p* = 0.002), compared to cattle with good body condition.

Housing condition also showed a strong association. Cattle kept under poor housing conditions had significantly higher odds of infestation (AOR = 10.54; 95% CI: 5.86–18.95; *p* < 0.001), whereas those under medium housing conditions also had increased odds (AOR = 2.58; 95% CI: 1.44–4.61; *p* = 0.001), compared to good housing conditions.

Herd management was significantly associated with infestation, where cattle kept in separated herds had lower odds of infestation compared to those in mixed herds (AOR = 0.60; 95% CI: 0.42–0.86; *p* = 0.005).

Breed type was also significant, as exotic breeds showed lower odds of infestation compared to crossbreeds (AOR = 0.60; 95% CI: 0.37–0.98; *p* = 0.042), whereas local breeds were not significantly different (AOR = 1.26; 95% CI: 0.86–1.84; *p* = 0.233).

Regarding owner‐related factors, basic literacy was associated with reduced odds of infestation (AOR = 0.55; 95% CI: 0.35–0.87; *p* = 0.011). However, sex of cattle (AOR = 1.02; *p* = 0.915), gender of household head (AOR = 1.02; *p* = 0.915) and administrative zone (AOR = 1.20; *p* = 0.608) were not significantly associated with tick infestation. Overall, tick infestation was mainly driven by body condition, housing quality, herd management and specific location effects.

**TABLE 7A:** Multivariable logistic regression analysis of animal‐related factors associated with tick infestation.

Risk factor	Category	NE	NP	Prevalence (%) [95% CI]	Adjusted OR [95% CI]	*p* value
Breed	Crossbred (Ref.)	276	108	39.1 [33.3, 45.2]	1.00	—
	Exotic	223	52	23.3 [17.9, 29.5]	0.60 [0.37, 0.98]	0.042
	Local	524	265	50.6 [46.2, 54.9]	1.26 [0.86, 1.84]	0.233
Sex (animal)	Female (Ref.)	532	207	38.9 [34.7, 43.1]	1.00	—
	Male	491	218	44.4 [40.0, 48.9]	1.02 [0.74, 1.41]	0.915
Body condition	Good (Ref.)	203	33	16.3 [11.4, 22.4]	1.00	—
	Medium	542	191	35.2 [31.1, 39.4]	2.12 [1.32, 3.41]	0.002
	Poor	278	201	72.3 [66.7, 77.4]	7.72 [4.58, 13.00]	<0.001
Housing condition	Good (Ref.)	183	18	9.8 [6.0, 15.1]	1.00	—
	Medium	390	102	26.2 [21.9, 30.9]	2.58 [1.44, 4.61]	0.001
	Poor	450	305	67.8 [63.3, 72.0]	10.54 [5.86, 18.95]	<0.001

Abbreviations: NE, number of animals examined; NP, number of positive animals; OR, odds ratio; Ref., reference category.

**TABLE 7B:** Multivariable logistic regression analysis of environmental and respondent‐related factors associated with tick infestation in cattle.

Risk factor	Category	NE	NP	Prevalence (%) [95% CI]	Adjusted OR [95% CI]	*p* value
Kebelle	Derg Ajen (Ref.)	129	42	32.6 [25.2, 40.9]	1.00	—
	Didba	233	86	36.9 [30.8, 43.3]	0.90 [0.50, 1.60]	0.711
	Genetie	159	76	47.8 [40.0, 55.6]	0.86 [0.46, 1.60]	0.625
	Mahbere Genet	170	72	42.4 [35.1, 50.0]	1.25 [0.69, 2.29]	0.460
	Meswati	38	16	42.1 [27.0, 59.1]	1.02 [0.39, 2.69]	0.962
	Tahtay Haya	60	40	66.7 [53.8, 77.5]	3.74 [1.62, 8.65]	0.002
	Tsibet	59	7	11.9 [5.6, 23.4]	0.26 [0.09, 0.72]	0.010
	Tsig'a	86	36	41.9 [31.6, 52.9]	0.53 [0.26, 1.07]	0.075
	Wargba	89	50	56.2 [45.3, 66.5]	0.66 [0.52, 1.64]	0.081
Herd type	Mixed (Ref.)	278	152	54.7 [48.9, 60.3]	1.00	—
	Separated	745	273	36.6 [33.1, 40.2]	0.60 [0.42, 0.86]	0.005
Zone	South‐eastern (Ref.)	532	200	37.6 [33.5, 41.6]	1.00	—
	Southern	491	225	45.8 [41.3, 50.3]	1.20 [0.60, 2.37]	0.608
Gender (respondent)	Female (Ref.)	433	202	46.7 [41.9, 51.4]	1.00	—
	Male	590	223	37.8 [33.9, 41.8]	1.02 [0.74, 1.41]	0.915
Education level	No education (Ref.)	527	248	47.0 [42.7, 51.3]	1.00	—
	Basic literacy	192	55	28.6 [21.8, 35.9]	0.55 [0.35, 0.87]	0.011
	Primary and junior	237	89	37.6 [31.5, 44.0]	0.83 [0.56, 1.23]	0.355
	Secondary and above	67	33	49.3 [36.8, 61.8]	1.41 [0.72, 2.77]	0.315

*Note*: The logistic regression model showed a good fit, as evidenced by a significant omnibus test (*χ* ^2^ (19) = 404.320, *p* < 0.001), acceptable pseudo‐*R* ^2^ values (Cox and Snell *R* ^2^ = 0.326; Nagelkerke *R* ^2^ = 0.44), and a non‐significant Hosmer–Lemeshow test (*p* = 0.145). The model correctly classified 75.4% of the 1023 cattle examined, with a sensitivity of 70.4% (correctly identifying 299 of 425 infested cattle) and a specificity of 78.9% (correctly identifying 472 of 598 non‐infested cattle). The area under the ROC curve (AUC) was 0.70, indicating acceptable discriminative ability. Overall, the model performed reasonably well in distinguishing infested from non‐infested cattle, demonstrating balanced accuracy and predictive power.

Abbreviations: CI, confidence interval; NE, number of animals examined; NP, number of positive animals; OR, odds ratio; Ref., reference category.

## 4 Discussion

### 4.1 Laboratory Tick Identification Results

Tick infestation remains one of the major ectoparasite challenges affecting cattle production in Ethiopia, with varying prevalence across different regions depending on ecological and management factors. The current study contributes to this growing body of evidence by documenting the overall prevalence of cattle tick infestation in the study area.

The distribution and abundance of tick species in Ethiopia have been widely reported by several researchers, highlighting the endemic nature of tick infestation across the country (Chumburo and Bayou 2021). In the current study, out of 1023 examined cattle, 425 were found infested with one or more tick species, resulting in an overall prevalence of 41.54%. This prevalence is in close agreement with earlier findings by Gebreselama et al. (2014), who reported a prevalence of 47.0% in Bishoftu, East Shewa Zone, and by Tamrat et al. (2023), who found a prevalence of 47.22% in Enarji Enawuga District, Eastern Gojjam Zone. Similarly, comparable prevalence rates were recorded by Zemene (2024) at 37.76% and Derara et al. (2020) at 36.6% in the Shashemene town. These similarities may be attributed to shared agroecological conditions, comparable livestock management systems and similar tick control practices.

On the other hand, lower prevalence rates have been documented in several regions, including a 22.9% prevalence across three districts in South Omo Zone (Getachew et al. 2024), 28.1% in Wayu Tuka (Fite 2023) and 28.28% in Kutaber Woreda (Wondmnew et al. 2018). These reduced prevalence levels may reflect more effective tick control measures, less favourable environmental conditions for tick survival or heightened community awareness and better access to veterinary services. Conversely, substantially higher prevalence rates have been reported in various other studies. For instance, Hadgu et al. (2018) reported 90% in Raya Azebo, Tigray; Seid and Mohammed (2017) and Gelelcha et al. (2019) recorded 96.4% in Chiro and Mizan Teferi, respectively. Likewise, Yenew et al. (2025) found a prevalence of 87.8% in Bahir Dar Zuria and Guanguwa, while other studies have documented rates of 71% (Tafesse and Amante 2019), 75.3% (Mekuriaw and Walelign 2016), 70.31% (Teshome et al. 2016) and 89.89% (Ayanaa et al. 2021). Werede and Afera (2014), Bedasso et al. (2014) and Admassu et al. (2015) also reported high prevalence rates of 86.1%, 84.1%, and 56.2%, respectively.

The substantial variability in tick prevalence across different regions likely stems from multiple interacting factors, including climatic and topographic differences, tick species composition, host density, veterinary service access, acaricide resistance and livestock management systems. The 41.54% prevalence identified in this study underscores the persistent threat ticks pose to cattle health and productivity, emphasizing the need for targeted and context‐specific control strategies.

#### 4.1.1 Genus‐Specific Tick Prevalence

##### 4.1.1.1 Prevalence of the *Rhipicephalus* Genus Ticks: Comparative Analysis

In the current study, the genus Rhipicephalus was the most dominant, with a prevalence of 75.9%. This figure is substantially higher than those reported in previous studies conducted in various parts of Ethiopia. For example, Tafesse and Amante (2019) reported a prevalence of 50.3% in Guto Gida district (Oromia), whereas Hadgu et al. (2018) found 54% in Raya Azebo district (Tigray). Lower prevalences were also recorded by Mekuriaw and Walelign (2016) (41.7%, Oromia), Alemu et al. (2014) (58.92%, Amhara), and Werede and Afera (2014) (3.5%, Tigray).

Statistically, the observed prevalence in the present study (75.9%) was significantly higher than the mean prevalence of 41.68% from previous studies (*χ* ^2^ = 464.49, *p* < 0.001). This marked difference may be attributed to regional ecological variations, seasonal timing of data collection, and differences in tick control practices, particularly the inconsistent or ineffective use of acaricides across locations.

##### 4.1.1.2 Prevalence of Hyalomma Genus Ticks: Comparative Analysis

The genus Hyalomma accounted for 21.7% of ticks in the present study. This prevalence closely aligns with the reports by Tafesse and Amante (2019) (20%) and Alemu et al. (2014) (20.33%). However, much higher prevalence was noted by Mekuriaw and Walelign (2016) in Arsi Zone (75.3%), whereas Hadgu et al. (2018) and Werede and Afera (2014) reported markedly lower prevalence rates of 2.7% and 1%, respectively. Compared to the average prevalence of 23.47% across prior studies, the present result did not differ significantly (*χ* ^2^ = 1.74, *p* > 0.05), suggesting a relatively consistent distribution of Hyalomma in various parts of Ethiopia under similar agro‐climatic conditions.

##### 4.1.1.3 Prevalence of Amblyomma Genus Ticks: Comparative Analysis

In the present study, the prevalence of Amblyomma spp. was recorded at 2.4%, which is relatively low compared to several previous findings across different regions of Ethiopia. For instance, a markedly higher prevalence was reported by Werede and Afera (2014) in the Werieleke district, Central Zone of Tigray, where 34.4% of the cattle examined were infested with Amblyomma ticks. Similarly, Tafesse and Amante (2019) reported 29.7% prevalence in Guto Gida district, East Wollega Zone, Oromia, and Alemu et al. (2014) found a prevalence of 20.74% in Dembia district, Gondar Zone, Amhara Region.

In contrast, findings from Hadgu et al. (2018) in Raya Azebo district, Southern Tigray, and Mekuriaw and Walelign (2016) in Sude Woreda, Arsi Zone, revealed Amblyomma prevalence rates closer to the current study, at 3% and 0.8%, respectively. These variations may be attributed to differences in ecological conditions, altitude, seasonal timing of sample collection, tick control practices and animal management systems.

Overall, the low Amblyomma prevalence observed in the current study might reflect localized environmental factors less favourable for this genus or could be the result of ongoing tick control measures, whereas the higher rates reported in other regions suggest more favourable conditions for Amblyomma proliferation or limited intervention strategies.

#### 4.1.2 Species‐Specific Tick Prevalence

Six Ixodid tick species were identified in the current study: R. evertsi evertsi, R. decoloratus, R. pulchellus, H. truncatum, H. marginatum rufipes and A. variegatum. Their prevalence varied, reflecting differences in environmental suitability, host exposure and control practices across the study area. These species are discussed individually below.

##### 4.1.2.1 Prevalence of *Rhipicephalus* (Boophilus) *evertsi evertsi*

The current study identified R. (Boophilus) evertsi evertsi in 26.2% of infested cattle, representing a moderate level of infestation. This prevalence closely aligns with findings by Mekuriaw and Walelign (2016), who reported 28.61% in Sude Woreda, Arsi Zone. However, lower prevalence rates have been observed in Bishoftu (15.4%) by Haramaya (14.66%) (Kassa and Yalew 2012) and Dembia (11.51%) by Alemu et al. (2014). Likewise, 16% was reported in Bahir Dar Zuria and Guanguwa (Yenew et al. 2025).

Much lower values were documented in Dassenech (7.54%) by Kemal and Abera (2017), Mizan‐Teferi (7.57%) by Gelelcha et al. (2019) and Yabello (3.36%) by Ayanaa et al. (2021), with the lowest being 0.43% in Borana Zone (Regassa 2001). Other low values include 2.35% in Guba‐Koricha (Ababa et al. 2017) and 5% in Wayu Tuka (Belete and Mekuria 2023). Conversely, a higher prevalence of 33.9% was recorded in Hawassa (Chumburo and Bayou 2021). These variations suggest that tick distribution is influenced by environmental suitability, host availability, tick control practices and seasonal factors across the different agro‐ecological zones.

##### 4.1.2.2 Prevalence of *Rhipicephalus* (Boophilus) *decoloratus*

In this study, R. (Boophilus) decoloratus was found in 25.9% of examined cattle, indicating moderate infestation. Comparable findings include 24.6% in Guto Gida District (Tafesse and Amante 2019) and 26.8% in Bedele District (Lemu et al. 2023). Similarly, the same authors reported 24.6% in a subsequent study in the same area.

However, higher prevalence levels have been documented elsewhere: 66.1% in Bahir Dar Zuria and Guanguwa (Yenew et al. 2025), 60.53% in Dassenech (Kemal and Abera 2017), 59.85% in Damot Gale (Detamo and Handalo 2020) and 40.86% in Dembia (Alemu et al. 2014). In contrast, lower prevalence was reported in Sude Woreda (17.72%), Bishoftu (11.7%), Yabello (7.01%) and as low as 2.9% in South Omo districts (Getachew et al. 2024). The lowest value was 1.6% in the Borana Zone (Regassa 2001). Such discrepancies could be due to ecological variations, tick control practices, and differences in host susceptibility and seasonality.

##### 4.1.2.3 Prevalence of *Rhipicephalus pulchellus*

R. pulchellus accounted for 23.8% of the ticks identified in this study. This prevalence is comparable to that reported in Dire Dawa (21.65%) by Jemal et al. (2022) and moderately lower than the 17.72% documented in Sude Woreda (Mekuriaw and Walelign 2016) and 10.6% in East Wollega Zone (Belete and Mekuria 2023). Significantly higher values were recorded in southern Ethiopia, particularly 73.17% in Yabello (Ayanaa et al. 2021) and 81.9% in the same area (Regassa 2001), suggesting that R. pulchellus thrives in hot, arid lowland environments. Similarly, 47.9% was reported in South Omo (Getachew et al. 2024) and 54% in Raya Azebo (Hadgu et al. 2018). Conversely, a very low prevalence of 1.04% was found in Ambo District (Gebremeskel et al. 2022), likely due to unfavourable climatic conditions or effective control practices. These findings emphasize the importance of localized tick ecology and adaptive tick management strategies.

##### 4.1.2.4 Prevalence of *Hyalomma truncatum*

In this study, H. truncatum represented 21.3% of the tick population, which is markedly higher than those reported in Dembia (7.36%; Alemu et al. 2014), Guto Gida (5.2%; Tafesse and Amante 2019) and Raya Azebo (2.7%; Hadgu et al. 2018). Even lower proportions were reported in Guba Koricha (2.4%; Ababa et al. 2017), Yabello (0.87%; Ayanaa et al. 2021) and across Borana Zone (0.56%; Regassa 2001). This increased presence may reflect regional ecological conditions favouring H. truncatum, limited acaricide use and the predominance of extensive grazing systems, as well as potential seasonal or climatic influences.

##### 4.1.2.5 Prevalence of *Amblyomma variegatum*

A. variegatum was identified in only 2.4% of the sampled cattle, a relatively low prevalence compared to studies conducted in Bahir Dar Zuria (50.3%; Yenew et al. 2025), Hawassa (43.2%; Chumburo and Bayou 2021) and Haramaya (38.87%; Kassa and Yalew 2012). Other moderate figures include 38.75% in Sude Woreda (Walelign and Mekuriaw and Walelign 2016) and 29.29% in Chiro (Seid and Mohammed 2017). In contrast, the current result aligns more closely with findings in Raya Azebo (3%; Hadgu et al. 2018), Borana (1.3%), and West Shewa (0.8%; Gebremeskel et al. 2022). These differences likely reflect ecological and seasonal factors as well as differences in tick control and surveillance programmes.

##### 4.1.2.6 Prevalence of *Hyalomma marginatum rufipes*

The prevalence of H. marginatum rufipes in this study was exceptionally low at 0.1%. This is considerably lower than the 5.44% reported in Sude Woreda (Mekuriaw and Walelign 2016), 4.7% in Bishoftu (Teshome et al. 2016) and 3% in Bahir Dar Zuria (Yenew et al. 2025). Other studies reported 2.6% in Hetosa (Hussain et al. 2021) and 0.27% in Yabello (Ayanaa et al. 2021). The minimal occurrence of this species may suggest unfavourable conditions for its establishment in the study area or effective tick control strategies that have limited its spread.

#### 4.1.3 Distribution of Ixodid Tick Genera by Sex and Infestation Patterns

In the present study, a total of 2952 adult Ixodid ticks were collected from cattle and categorized into three genera: Rhipicephalus (75.9%), Hyalomma (21.7%) and Amblyomma (2.4%). Across all genera, male ticks outnumbered females, with an overall female‐to‐male (F:M) ratio of 1:2.77. This male dominance was most pronounced in Hyalomma (1:6.36), followed by Amblyomma (1:4.83) and Rhipicephalus (1:2.27). These findings suggest genus‐specific variations in sex distribution, potentially reflecting behavioural or ecological differences such as longer host‐seeking or feeding periods in males.

The predominance of Rhipicephalus ticks in the current study is consistent with several reports from different parts of Ethiopia. Yalew et al. (2017) in and around Hararghe town, Eastern Ethiopia, also found higher proportions of Rhipicephalus (556 males vs. 208 females; F:M = 1:2.67) and Amblyomma (402 males vs. 280 females; F:M = 1:1.43), supporting the trend of male predominance.

Similarly, Esayas (2024) reported a substantial male‐biased distribution in Amblyomma (1609 males vs. 477 females; F:M = 1:3.37) and Rhipicephalus (516 males vs. 316 females; F:M = 1:1.63) in the Jimma region. Bedasso et al. (2014) also supported the trend of male predominance with male‐to‐female ratio of Rhipicephalus 2.3:1 (4167/1783), *Amblyomma* 2.6:1 (899/346) and Hyalomma spp. 3.4:1 (188/55).

Conversely, Wondimu and Bayu (2021) reported a higher number of female Rhipicephalus (73 females vs. 44 males; F:M = 1.66:1), which contrasts with most other studies, including the present one. This variation might be attributed to differences in sampling period, host physiology, tick seasonality or microclimatic conditions across regions. Infestation pattern analysis revealed that single‐species infestations were more common across all genera, especially in Rhipicephalus (782 cases), indicating its ability to establish dominant infestations without competition.

However, Hyalomma was frequently found in mixed‐species infestations (236 cases), suggesting ecological flexibility and potential co‐occurrence with other genera on the same host. Overall, the observed sex distribution and infestation patterns emphasize the ecological dominance of Rhipicephalus in the region and underscore the importance of targeted tick control strategies considering genus‐ and sex‐specific dynamics.

#### 4.1.4 Attachment Site Distribution of Tick Species Infesting Cattle

The present study demonstrated variation between farmers’ perceptions and empirical observations regarding tick attachment sites. Based on the questionnaire survey, respondents most frequently identified the teat/udder (30.2%), tail/head (22.6%) and lateral/ventral regions (18.9%) as common sites of infestation. These areas are highly visible during daily management and of economic concern, particularly the udder due to its impact on milk production.

However, laboratory and observational results indicated more species‐specific attachment patterns. The lateral/ventral region and dewlap/brisket were the principal predilection sites for R. pulchellus (34.4% and 31.6%), R. evertsi evertsi (32.6% and 30.7%) and H. truncatum (41.9% and 33.4%). A. variegatum exhibited a strong preference for the teat/udder (39.8%), whereas R. decoloratus was more evenly distributed, with higher proportions on the tail/head (24.6%) and teat/udder (17.7%). H. rufipes was less abundant overall, with a slight preference for the dewlap/brisket and lateral/ventral regions. These patterns reflect the influence of host‐related factors such as grooming, skin thickness and blood vessel distribution, in addition to species‐specific behaviour.

Comparison with previous studies reveals both consistency and divergence. Similar to the current findings, Yalew et al. (2017) and Dabasa et al. (2017) reported high infestations of Amblyomma species on the udder, scrotum, and perineum, whereas studies by Tadesse et al. (2012) and Regassa (2001) highlighted the neck, groin, and dewlap as frequent sites. Namgyal et al. (2021), in Bhutan, also reported predilection for the neck and groin. Such variation across studies likely reflects ecological differences, host factors, and the composition of tick species in different regions.

### 4.2 Comparison of the Present Study With Previous Reports From Tigray

In this study, 1023 cattle were examined, of which 425 were infested with ticks, giving an overall tick prevalence of 41.54%. A total of 2952 adult ticks were collected and identified into three genera: Rhipicephalus (75.9%), Hyalomma (21.7%) and Amblyomma (2.4%). The predominant tick species identified were R. evertsi evertsi (26.2%), R. decoloratus (26.0%) and R. pulchellus (23.8%).

This prevalence is lower than that reported among Raya cattle by Hadgu et al. (2018), who recorded a prevalence of 90.0% (405/448) in Raya‐Azebo district, and Werede and Afera (2014), who documented a prevalence of 86.1% (603/700) in Begait cattle in Werieleke. Moreover, Hailu et al. (2024) identified nine tick species among 384 cattle, with Hyalomma species (55.69%) predominating.

The markedly higher prevalence rates in these studies compared with the present findings may be attributable to differences in host breed susceptibility, production and management systems, agro‐ecological variation, seasonal timing of sampling, and the level of tick control practices implemented. In contrast, studies conducted on small ruminants have reported variable but sometimes comparable prevalence estimates. For example, Degefa et al. (2018) observed a prevalence of 44.7% (137/306) in goats and 29.5% (23/78) in sheep in Enderta District, yielding an overall prevalence of 41.6% (160/384).

Regarding the composition of tick genera, the predominance of Rhipicephalus spp. (75.9%) in the present study is consistent with the observations of Degefa et al. (2018), who reported Rhipicephalus spp. at 62.6%. However, this contrasts with Werede and Afera (2014), who reported Amblyomma spp. (75.6%) as the dominant genus among 700 Begait cattle, and Hailu et al. (2024), who identified Hyalomma spp. (55.69%) as the predominant genus across sites in Mekelle, Workamba and Hashenge. Such variation may be linked to ecological differences, vector adaptation patterns and host preferences.

At the species level, the detection of six tick species in the current study accords with Hadgu et al. (2018), who similarly identified six species belonging to *Rhipicephalus*, *Amblyomma*, Boophilus and Hyalomma in Raya‐Azebo. By contrast, several other studies in the region characterized ticks only to the genus level, thereby limiting detailed species‐level comparison.

Collectively, the findings of the present study reaffirm that Rhipicephalus spp. are the principal ticks infesting cattle in Tigray, although the relative proportions of *Amblyomma* and *Hyalomma* spp. vary by district, host species, and management system. Differences in agro‐ecology, host genetics, tick control practices and production environments are likely to account for the observed heterogeneity in both prevalence and species composition across studies.

### 4.3 Univariable and Multivariable Logistic Regression Analysis

#### 4.3.1 Comparison of Univariable Logistic Regression Results With Previous Studies

In this study, significant associations were found between tick infestation and various animal and management‐related factors, such as kebeles, breed, and BCS, housing condition, and herd type; however, sex and age had weaker or non‐significant effects. These findings are largely in line with previous studies conducted in different regions of Ethiopia, although some differences were observed. A notable geographic variation in infestation risk was identified, with cattle from Tahtay‐Haya and Wargba showing significantly higher odds of infestation compared to those from Derg Ajen.

Cattle from Tsibet, on the other hand, exhibited lower risk. Similar kebeles‐level differences were reported by Belete and Mekuria (2023) in Slamago district, Kebede et al. (2025) in Guder town, and Zemene (2024) in Lay Armachiho, where ecological and grazing system variations were identified as key factors influencing infestation risk. Furthermore, breed was identified as an important predictor in this study. Local cattle were more susceptible to infestation compared to crossbred cattle, whereas exotic breeds showed a protective effect against tick infestation.

These findings are in line with previous studies, such as those by Bedasso et al. (2014) in the Borana Zone and Wegi (2023) in Ejere District, which reported higher infestation burdens in indigenous zebu cattle managed under extensive systems. Similar results were also reported by Yenew et al. (2025) in Northwest Ethiopia, reinforcing the role of breed in influencing infestation risk. Although male cattle showed a tendency for higher infestation odds in this study, the effect did not reach significance.

This is consistent with the findings of Zemene (2024), who also reported non‐significant sex difference, but contrasts with the results of Abdurrahman et al. (2022) in East Hararghe, who observed significantly higher infestation prevalence in males, likely due to their increased exposure during grazing and draft work. In contrast, age was not statistically associated with infestation in the current study, which is in line with findings of Kebede et al. (2025) and Yenew et al. (2025). However, Belete and Mekuria (2023) reported higher infestation rates in older animals, which may reflect cumulative exposure over time.

Body condition was identified as one of the most significant predictors in this study, with cattle in poor body condition being more than 13 times more likely to be infested compared to those in good condition. This finding aligns with previous research by Wegi (2023), Abdurrahman et al. (2022), Dabasa et al. (2017) and Zemene (2024), all of which reported higher tick burdens in animals with poor body condition, primarily due to reduced immunity and grooming ability.

Housing condition also plays a critical role; cattle housed in poorly maintained environments were nearly 20 times more likely to be infested than those in well‐maintained housing. This is consistent with the findings of Belete and Mekuria (2023) and Abdurrahman et al. (2022), who highlighted that poor sanitation and structural cracks in housing contribute to the proliferation of tick populations.

Additionally, herd type was found to be a significant factor, with animals in mixed herds showing higher infestation rates compared to those in separated herds. This is consistent with the findings of Kebede et al. (2025), who noted that communal grazing and mixed herd management enhance tick transmission through increased animal contact.

Overall, the current result supports findings from various regions in Ethiopia, particularly regarding the influence of breed, body condition, housing and herd management. However, some differences in the effects of sex and age may be attributed to variation in management practices, ecological conditions and sampling methods across different areas.

#### 4.3.2 Comparison of Multivariable Regression Findings on Risk Factors for Tick Infestation With Previous Studies

The present study revealed that cattle body condition, housing condition; breed, kebeles, herd type and owner education level were significant predictors of tick infestation. Poor body condition markedly increased infestation risk; supporting earlier studies in Ethiopia that reported emaciated animals are more vulnerable due to weakened immunity and reduced grooming capacity (Admassu et al. 2015; Dabasa et al. 2017; Mathewos et al. 2021).

Housing condition was also strongly associated, with poorly maintained housing increasing odds more than tenfold, in agreement with Waktole et al. (2018) and Yalew et al. (2017).

Breed differences were evident, where exotic cattle were less infested than crossbreds, contrasting with Adugna and Tamrat (2022) and Mathewos et al. (2021), who found local breeds more heavily infested. These variations may relate to differences in grazing systems, breed resilience and local tick ecology.

Environmental and management factors also played a key role. Significant spatial variation was noted across kebeles, with higher infestation in Tahtay Haya and lower odds in Tsibet, reflecting the microclimatic and ecological heterogeneity previously reported in Ethiopia and Rwanda (Zemene 2024; Gabriel et al. 2025). Separated herds were less affected than mixed herds, confirming the role of communal grazing in facilitating tick transmission (Dabasa et al. 2017; Waktole et al. 2018).

Owner education showed a protective effect, where basic literacy reduced infestation risk, consistent with Abdurrahman et al. (2022) and Yenew et al. (2025), whereas Belete and Mekuria (2023) and Kebede et al. (2025) did not find education significant, suggesting that its impact depends on veterinary extension and awareness programmes.

Collectively, these findings emphasize the multifactorial nature of tick infestation in cattle, where both biological vulnerability (body condition and breed) and management‐environmental factors (housing, herd type, education and location) interact to shape risk.

Overall comparison: Taken together, the present study corroborates the consensus from several Ethiopian studies (Admassu et al. 2015; Dabasa et al. 2017; Waktole et al. 2018; Abdurrahman et al. 2022; Yenew et al. 2025; Belete and Mekuria 2023) that poor body condition, poor housing, breed susceptibility and management practices are key independent determinants of tick infestation. However, variations exist in the role of breed and education, reflecting ecological and management differences between regions. These findings highlight the multifactorial nature of tick infestation, where both biological vulnerability (body condition, breed) and environmental‐management factors (housing, herd type, kebeles and education) interact to shape risk profiles.

## 5 Conclusion and Recommendation

The study demonstrated that tick infestation in cattle is widespread in the three districts of Tigray, with an overall prevalence of 41.5%. The infestation was dominated by *Rhipicephalus* species, followed by *Hyalomma* and *Amblyomma*. Species exhibited distinct attachment site preferences, with *Amblyomma* concentrated on the udder and *Rhipicephalus* and *Hyalomma* favouring the brisket and ventral regions. Tick prevalence was significantly influenced by animal factors (body condition and breed), management practices (housing and herd type) and owner literacy.

### 5.1 Recommendations

- Targeted Tick Control: Implement control strategies focusing on species‐specific predilection sites, particularly the udder, brisket, and ventral areas, to maximize the effectiveness of inspection and acaricide application.
- Improved Animal Management: Enhance cattle housing and separate herd management where feasible, and maintain good body condition to reduce tick susceptibility.
- Farmer Education: Increase community awareness programmes emphasizing tick‐borne disease risks and proper control practices, integrating both scientific and traditional knowledge.
- Integrated Control Strategies: Combine acaricide use with cultural practices and, where appropriate, ethno veterinary remedies to promote sustainable and cost‐effective tick management.

## Author Contributions

Girmay Hiluf Asefa and Mebrahtom Gebre Libanos Hiben contributed to the conceptualization of the study. The study methodology was developed by Girmay Hiluf Asefa, Mebrahtom Gebre Libanos Hiben, Yohannes Tekle Asfaw and Nigus Abebe Shumye. Supervision was provided by Mebrahtom Gebre Libanos Hiben, Yohannes Tekle Asfaw and Nigus Abebe Shumye throughout the study. Field investigation data collection was undertaken by Girmay Hiluf Asefa and Melesew Adisu Abrha. Tick identification and curation was performed by Girmay Hiluf Asefa with supervisory impute from Nigus Abebe Shumye. The original draft was prepared by Girmay Hiluf Asefa and the manuscript was critically reviewed and edited by Mebrahtom Gebre Libanos Hiben, Yohannes Tekle Asfaw and Nigus Abebe Shumye.

## Ethics Statement

This study received ethical clearance from the Institutional Review Board (IRB) at Mekelle University College of Health Sciences (reference no.: MU‐IRB 2674/2025). Local officials and potential participants were briefed on the aims and rationale of the research. Participation was completely voluntary, and all respondents gave informed consent before taking part in the data collection process. Strict confidentiality and anonymity were upheld throughout the study, with no personally identifiable information being collected.

## Consent

All participants were informed about the objectives of the study and provided their voluntary consent prior to participation.

## Conflicts of Interest

The authors declare no conflicts of interest.

## Presentation Work

Preliminary result of this study was presented at the research review day as a poster presentation and abstract at Mekelle University Research review day.

## Supporting information




**Figure** **S1**: Spatial distribution of sampling locations for tick species in Enderta, Endamehoni, and Raya Azebo districts.
**Figure S2**: Bar chart showing the prevalence of cattle tick infestation across selected animal, management and owner related risk factors in the study area.
**Figure S2.1**: Prevalence of cattle tick infestation by altitude.
**Figure S2.2**: Prevalence of cattle ticks by Breed.
**Figure S2.3**: Prevalence of cattle ticks by body Conditions.
**Figure S2.4**: Prevalence of cattle ticks by Management System.
**Figure S2.5**: prevalence of cattle Ticks by herd Type.
**Figure S2.6**: Prevalence of cattle ticks by housing Conditions.
7. ANNEXES

## Data Availability

The data supporting this study are available from the corresponding author up on reasonable request.
