# Supplementary material for: Household‐Based Epidemiology of Bovine Ixodid Tick Infestation and Associated Risk Factors in Endamehoni, Raya‐Azebo and Enderta Districts, Tigray Region, Northern Ethiopia
Source: Vet Med Sci. 2026 Aug 5;12(5):e71095. doi: 10.1002/vms3.71095 (PMC13439525; doi:10.1002/vms3.71095)
Supplement: Supplementary file 1 [file VMS3-12-e71095-s001.docx]

Figure 1. Spatial distribution of sampling locations for tick species in Enderta, Endamehoni, and Raya Azebo districts

Figure 2: Bar chart showing the prevalence of cattle tick infestation across selected animal, management and owner related risk factors in the study area.

Figure 2.1: prevalence of cattle tick infestation by altitude

Figure 2.2: Prevalence of cattle ticks by Breed

Figure 2.3: Prevalence of cattle ticks by body Conditions

Figure 2.4: Prevalence of cattle ticks by Management System

Figure 2.5: prevalence of cattle Ticks by herd Type

Figure 2.6: Prevalence of cattle ticks by housing Conditions

**7. ANNEXES**

**Annex1: Field and Laboratory Data Collection Formats**

| Id. No. | District | Kebele | Breed | Sex | Age | BCS | Presence of tick | Site of attachment |
| --- | --- | --- | --- | --- | --- | --- | --- | --- |
|  |  |  |  |  |  |  |  |  |
|  |  |  |  |  |  |  |  |  |
|  |  |  |  |  |  |  |  |  |
|  |  |  |  |  |  |  |  |  |
|  |  |  |  |  |  |  |  |  |
|  |  |  |  |  |  |  |  |  |
|  |  |  |  |  |  |  |  |  |
|  |  |  |  |  |  |  |  |  |
|  |  |  |  |  |  |  |  |  |
|  |  |  |  |  |  |  |  |  |
|  |  |  |  |  |  |  |  |  |
|  |  |  |  |  |  |  |  |  |
|  |  |  |  |  |  |  |  |  |
|  |  |  |  |  |  |  |  |  |
|  |  |  |  |  |  |  |  |  |
|  |  |  |  |  |  |  |  |  |
|  |  |  |  |  |  |  |  |  |
|  |  |  |  |  |  |  |  |  |
|  |  |  |  |  |  |  |  |  |
|  |  |  |  |  |  |  |  |  |
|  |  |  |  |  |  |  |  |  |
|  |  |  |  |  |  |  |  |  |
|  |  |  |  |  |  |  |  |  |

**Annex 2:** **Format for Data Collection for Tick Identification**

| Id. No. | Tick count | Tick genus | Tick species | Tick sex | Tick diversity |
| --- | --- | --- | --- | --- | --- |
|  |  |  |  |  |  |
|  |  |  |  |  |  |
|  |  |  |  |  |  |
|  |  |  |  |  |  |
|  |  |  |  |  |  |
|  |  |  |  |  |  |
|  |  |  |  |  |  |
|  |  |  |  |  |  |
|  |  |  |  |  |  |
|  |  |  |  |  |  |
|  |  |  |  |  |  |
|  |  |  |  |  |  |
|  |  |  |  |  |  |
|  |  |  |  |  |  |
|  |  |  |  |  |  |
|  |  |  |  |  |  |
|  |  |  |  |  |  |
|  |  |  |  |  |  |
|  |  |  |  |  |  |
|  |  |  |  |  |  |
|  |  |  |  |  |  |
|  |  |  |  |  |  |

**Annex 3: Materials and Chemicals**

**Table in the Appendix 4: Materials and Chemicals**

| Materials | Distil water |
| --- | --- |
| Permanent marker | Ethanol 70% |
| Loops |  |
| Petri dish |  |
| Sampling bottle |  |
| Stereomicroscope |  |
| Blunt forceps |  |
| Gohan |  |
| Pen |  |
| Paper |  |
| Glove |  |
| Samsung Mobile M-14 (Camera) |  |

**Annex 4: Cattle Age Determination Based on Dentition**

| Age of cattle | Description |
| --- | --- |
| About less than a year | Full set of deciduous incisors |
| 1-2 years | Two or more the temporary incisor teeth present. |
| 3 years | Permanent first intermediates, one on each side of the pinchers, are cut. Usually those are fully developed at three years |
| 3 and ½ yeas | The second intermediates or laterals are cut. They are on a level with the first intermediates and begin to wear at four years. |
| 4 and ½ years | The corner teeth are replaced. At five years the animal usually has the full complement of incisors with the corners fully developed. |
| 5- 6 years | The permanent pinchers are leveled, both pairs of intermediates are partially leveled, and the corner incisors show wear. |
| 7-10 years | At seven or eight years, the pinchers show noticeable wear; at eight or nine years, the middle pairs show noticeable wear; and at 10 years, the corner teeth show noticeable wear. |
| Above 10 years | After the animal passes the sixth year, the arch gradually loses its rounded contour and becomes nearly straight by the 12^th^ year. In the meantime, the teeth gradually become triangular in shape, distinctly separated, and show progressive wearing to stubs. Those conditions become more marked with increasing age. |

**Source:** Ethiopian Ministry of Agriculture (2024)

**Annex 5: Protocol for Evaluating Body Condition Scores**

| Score | Condition | Description |
| --- | --- | --- |
| 1 | P- | Severe emaciation; minimal body condition with pronounced skeletal structure and lack of visible musculature |
| 2 | P | Moderate emaciation; ribs and transverse processes are distinctly projected, and the animal appears underweight. |
| 3 | P+ | Mild emaciation; individual dorsal spines are pointed and palpable, with visible musculature along the hips, tail, and head. |
| 4 | M- | Moderate condition; ribs, hips, and dorsal spines are clearly visible, with a slight concavity between the ribs and moderate subcutaneous fat above the back |
| 5 | M | Moderate condition; ribs are visible, with minimal fat cover over the dorsal spines. The animal appears well muscled but not excessively fat. |
| 6 | M+ | Good condition; Dorsal spines are smooth, well covered, and easily palpable. The animal has balanced body condition with adequate muscle mass and fat cover. |
| 7 | G- | Good condition; dorsal spines are smooth, and well covered with minimal fat deposition. The animal is firm to touch but lacks sharpness in the spine. |
| 8 | G | Good condition; fat cover is present in critical areas, such us the transverse process, but is not excessive. The animal appears healthy and well nourished. |
| 9 | G+ | Excellent condition; fat deposition is Heavey and visible apparent on the tail, head, brisket, dorsal, spines, ribs, and hook. |

Note: body condition scores; 1,2 and 3 are classified as poor body condition scores; 4,5 and 6 are considered medium body condition; and 7, 8, and 9 are categorized as good body condition.

**Source:** Nicholson and Butterworth (1986)

**Annex 6: Tick Identification Species Distinguishing Features of Adult Ixodid Ticks**

**1. Amblyomma variegatum**

- Mouth parts very long, elongate 2nd segment of palps and banded legs.
- Consecutum and scutum ornate, male consultum is dark orange.
- Their Eyes are bready and festoons are present, festoons in males are uniformly dark.
- Three host ticks.

2**. Rhipicephalus (Boophilus) decoloratus**

- Mouth parts very short and dentition on hypostome is arranged in two columns each consisting
- Of numerous rows each of which consists three denticles 3/3 dentition.
- The basis capituli is hexagonal in shape.
- The Conscutum in male is yellowish in color and often so poorly sclerotized that the outlines of the gut can be seen through it.
- There are numerous hairs on the conscutum of males and the scutum of females.
- Eyes present but not conspicuous and no festoons
- Adanal plates and accessory adanal plates of males well developed, caudal process presents in Males,
- They are one host ticks.

**3. Rhipicephalus pulchellus**

- It is a medium sized Rhipicephalus species with striked dark brown and an ivory-colored pattern on the conscutum while the scutum of female is ivory colored except brown patches around the eyes.
- Mouth parts short, basis capituli generally hexagonal in shape.
- The eyes are flat and Festoons present.
- The adanal plates are fairly long and engorged males a caudal appendage is present.
- In males the legs increase in size from I to IV.
- Majority is three host ticks, but a few are two host ticks.

**5. Hyalomma marginatum rufipes**

- Mouth parts long, 2ndsegment of palps elongate.
- Dark brown to nearly black conscutum of male is broadly oval and the entire surface is covered with medium sized coarse punctuations.
- The brown legs are brightly banded with ivory-colored rings. The brown legs are brightly banded with ivory-colored rings.
- Eyes present and convex and Festoons present.
- The adanal have square ends and subanal plates are small and aligned with adanal plates.
- The genital apron of female is convex; the genital aperture is very broadly V shaped.
- Two or three host ticks. Source for all species: (Walker et al., 2003)

**Annex7: Figure of Tick Species Identified in the Laboratory**

**Mixed tick species**
